# Coronary venous lead reimplantation vs. left bundle branch area pacing crossover following cardiac resynchronization therapy defibrillator extraction: a single-centre experience

**DOI:** 10.1093/europace/euae101

**Published:** 2024-04-26

**Authors:** Matteo Baroni, Alberto Preda, Marco Carbonaro, Matteo Fortuna, Fabrizio Guarracini, Lorenzo Gigli, Patrizio Mazzone

**Affiliations:** De Gasperis Cardio Center, Electrophysiology Unit, Niguarda Hospital, Piazza dell’Ospedale Maggiore 3, 20162 Milan, Italy; De Gasperis Cardio Center, Electrophysiology Unit, Niguarda Hospital, Piazza dell’Ospedale Maggiore 3, 20162 Milan, Italy; De Gasperis Cardio Center, Electrophysiology Unit, Niguarda Hospital, Piazza dell’Ospedale Maggiore 3, 20162 Milan, Italy; De Gasperis Cardio Center, Electrophysiology Unit, Niguarda Hospital, Piazza dell’Ospedale Maggiore 3, 20162 Milan, Italy; De Gasperis Cardio Center, Electrophysiology Unit, Niguarda Hospital, Piazza dell’Ospedale Maggiore 3, 20162 Milan, Italy; De Gasperis Cardio Center, Electrophysiology Unit, Niguarda Hospital, Piazza dell’Ospedale Maggiore 3, 20162 Milan, Italy; De Gasperis Cardio Center, Electrophysiology Unit, Niguarda Hospital, Piazza dell’Ospedale Maggiore 3, 20162 Milan, Italy

**Keywords:** Transvenous lead extraction, Left bundle branch area pacing, Cardiac resynchronization therapy, Heart failure

## Introduction

The steady growth of conduction system pacing (CSP) paved the way to new anti-bradycardia therapies, preserving intracardiac synchrony and yielding positive effects on cardiac contractility.^1^ Cardiac resynchronization therapy (CRT) consistently demonstrated benefits in a wide spectrum of heart failure conditions.^2^ Currently, CRT via left ventricular epicardial pacing (EP) using a coronary sinus (CS) lead is the recommended standard of care, with CSP considered as a bailout strategy.^3^ Nevertheless, there are several concerns of EP such as unpredictable CS branches anatomy, lead stability, diaphragmatic stimulation, and time-consuming procedure. After transvenous lead extraction (TLE) of a CRT system, EP replacement may be very challenging due to CS adherences, remnants, and venous branch occlusion,^4^ leading to longer procedures, increased risk of infection, and lower probability to achieve an optimal outcome. In this context, CSP emerges as a promising alternative to EP,^5^ in particular through left bundle branch area pacing (LBBAP).^6^ The aim of this study was to explore the feasibility and safety of LBBAP as an alternative to EP in patients with absence or unfavourable anatomy of posterolateral CS branches following TLE of a conventional CRT defibrillator (CRT-D).

## Methods

This prospective, single-centre study enrolled consecutive patients admitted to our department for TLE and reimplantation of a CRT-D device from July 2022 to April 2023. All procedures were carried out in our electrophysiology laboratory with on-site cardiac surgery backup.^6^ Transvenous lead extraction was performed following a standardized stepwise approach, as previously described,^7^ involving transition from simpler to more complex techniques. In cases of lead malfunction, TLE and reimplantation were performed as part of the same procedure. In patients with active infection, the timing of CRT-D reimplantation was determined according with the consultant infectivologist after a variable time of antibiotic therapy. In such instances, the skin-to-skin and fluoroscopic times of both procedures were summed. The decision between EP and LBBAP strategy was related to the presence of visible posterolateral CS branches at post-TLE CS venography. Left bundle branch area pacing with a stylet-driven lead (Solia S60, Biotronik, Germany) was therefore performed when posterolateral CS branches were absent or exhibited unfavourable anatomy. Data of interest included EP/LBBAP effective stimulation, skin-to-skin time, duration of paced QRS complex, and electrical EP/LBBAP lead parameters. Results were compared between patients undergoing LBBAP and EP reimplantation. Data are presented as mean ± standard deviation or frequencies of patients. Parametric continuous variables were assessed using the unpaired *t*-test, while non-parametric continuous variables were analysed using the Mann–Whitney *U* test. Categorical variables were subjected to the Pearson *χ*^2^ test. Statistical significance was set at *P* < 0.05. The study received approval from the competent Ethics Committee.

## Results

The study cohort included 26 patients (mean age of 68.3 ± 7.2 years, 12% females). Population characteristics are summarized in *Table 1*. Reasons for TLE included pocket infection (*n* = 11, 42%), lead malfunction (*n* = 8, 31%), and endocarditis (*n* = 7, 27%). Seven patients (27%) underwent multiple procedures. Mean lead dwell time was 88.9 ± 10 months. Seventeen patients (65%) had an estimated high procedural risk according to ELECTRa Registry Outcome Score (EROS) score.^8^ Successful TLE was achieved in 25 patients (96%) with the extraction of 80 leads, including 2 abandoned right ventricle leads. All patients affected by endocarditis and pocket infection, as well as six patients (75%) with lead malfunction, underwent contralateral reimplantation (100% on the right side) due to a high risk of infection or vascular unavailability. In the remaining two patients that needed preserved magnetic resonance imaging compatibility, a complete ipsilateral replacement was performed. There were two (7.7%) TLE-related pericardial effusion without the need for medical intervention. Overall, 12 (46%) LBBAP and 14 (54%) EP were reimplanted. Both strategies achieved effective LBBAP/EP stimulation in all cases. Left bundle branch area pacing reimplantation resulted in a quicker procedure (109.5 ± 34.2 vs. 126.5 ± 46.8 min, *P* < 0.001), reduced radiologic exposure (877.0 ± 234.6 vs. 1332.0 ± 515.1 s, *P* < 0.001), and lower ventricular pacing thresholds (0.7 ± 0.1 V vs. 1.4 ± 0.6 V, *P* < 0.001) compared to EP. Despite similar pre-TLE paced QRS complex durations (127 ± 14 ms vs. 133 ± 14 ms, *P* = 0.610), LBBAP resulted in a decreased QRS duration compared to EP via the newly implanted CS lead (105 ± 13 ms vs. 127 ± 10 ms, *P* < 0.001). No periprocedural complications related to LBBAP or the newly implanted CS lead were observed.

**Table 1:** Clinical characteristics and procedural features of the study population

	Total (*n* = 26)	LBBAP (*n* = 12)	EP (*n* = 14)	*P*-value
Age at extraction (years)	68.3 ± 9.2	66.4 ± 5.1	69.7 ± 8.2	0.538
Female (*n*, %)	3 (12%)	1 (8%)	2 (14%)	0.859
Indication to extraction				
Endocarditis	7 (27%)	4 (33%)	3 (21%)	0.362
Lead malfunction	8 (31%)	3 (25%)	5 (36%)	0.573
Pocket infection	11 (42%)	5 (42%)	6 (43%)	0.758
Height (cm)	169.7 ± 6.4	169.4 ± 9.8	170.5 ± 5.2	0.757
Weight (kg)	71.6 ± 8.1	72.5 ± 14.0	71.1 ± 11.7	0.811
Smoking history (*n*, %)	11 (42%)	5 (42%)	6 (43%)	0.856
Hypertension (*n*, %)	16 (62%)	8 (67%)	8 (57%)	0.761
Diabetes mellitus (*n*, %)	5 (19%)	2 (17%)	3 (21%)	0.882
Chronic kidney disease (*n*, %)	10 (38%)	4 (33%)	6 (43%)	0.732
Cardiac disease (*n*, %)				
Dilated cardiomyopathy	12 (46%)	5 (42%)	7 (50%)	0.673
Ischaemic cardiomyopathy	8 (31%)	5 (42%)	3 (21%)	0.265
Hypertrophic cardiomyopathy	3 (11%)	1 (8%)	2 (14%)	0.781
Valvular cardiomyopathy	3 (11%)	1 (8%)	2 (14%)	0.678
Multiple CIED procedures (*n*, %)	7 (27%)	3 (25%)	4 (29%)	0.591
Time to first CIED procedure to extraction (months)	88.9 ± 10	87.5 ± 8	91 ± 7	0.227
Previous cardiac surgery (*n*, %)	7 (27%)	2 (17%)	5 (36%)	0.823
HAS-BLED score	1.3 ± 0.7	1.4 ± 0.8	1.3 ± 0.5	0.691
EROS score >1	17 (65%)	7 (58%)	10 (71%)	0.783
LVEF (%)	40.6 ± 8.1	39.2 ± 4.5	41.4 ± 7.6	0.559
Pre-operative tricuspid regurgitation >2+ (*n*, %)	4 (15%)	2 (17%)	2 (14%)	0.672
Postoperative tricuspid regurgitation >2+ (*n*, %)	6 (23%)	3 (25%)	3 (21%)	0.532
Concomitant extraction and reimplantation	18 (69%)	8 (67%)	10 (71%)	0.891
Total lead extracted				
Right atrium	26	12	14	0.987
Right Ventricle	28	13	15	0.961
Left ventricle	26	12	14	0.987
Extraction type				
Manual traction	13 (16%)	4 (11%)	9 (21%)	0.152
Locking stylets	32 (40%)	17 (46%)	15 (35%)	0.313
Mechanical extractors	35 (44%)	16 (43%)	19 (44%)	0.917
Snare	2 (2%)	2 (5%)	0	
Skin-to-skin time (min)	119.4 ± 49.2	109.5 ± 34.2	126.5 ± 46.8	**<0.001**
Radiology exposure (s)	1104 ± 494.6	877.0 ± 234.6	1332.0 ± 515.1	**<0**.**001**
Complete extraction (*n*, %)	25 (96%)	11 (92%)	14 (100%)	–
Procedural complications (*n*, %)	2 (8%)	1 (8%)	1 (7%)	0.956
Pre-operative QRS time (ms)	130 ± 12.6	127.4 ± 13.8	133.0 ± 14.4	0.610
Postoperative QRS (ms)	115.4 ± 12.1	105.0 ± 13.1	127.1 ± 10.4	**<0**.**001**
Delta QRS time (ms)	− 8.33 ± 4.2	− 10.9 ± 7.5	− 6.8 ± 10.1	**<0**.**001**
Ventricular sensing (mV)	12.7 ± 5.4	11.6 ± 2.4	14.6 ± 3.6	0.297
Ventricular pacing impedance (ohm)	769.7 ± 291.5	753.3 ± 140.9	781.8 ± 340.1	0.858
Ventricular pacing threshold (V)	1.1 ± 0.5	0.7 ± 0.1	1.4 ± 0.6	**<0**.**001**
Ventricular pacing threshold (ms)	0.6 ± 0.2	0.5 ± 0.1	0.7 ± 0.3	0.869

*P*-values demonstrating statistically significant variances are highlighted in bold.

CIED, cardiac implantable electronic device; EP, epicardial pacing; LBBAP, left bundle branch area pacing; LVEF, left ventricle ejection fraction.

## Discussion

**Figure 1:**
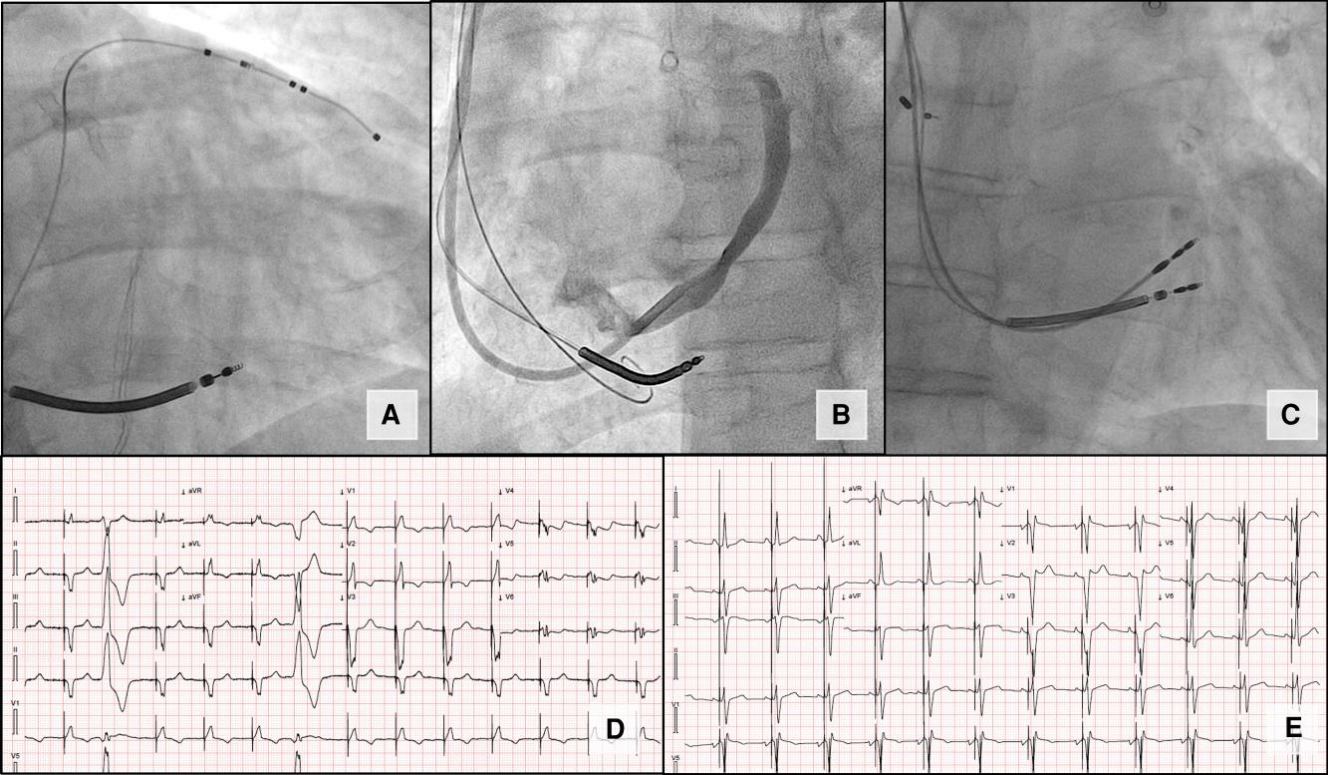
Example of CRT-D to LBBAP crossover. (*A*) Pre-TLE position of the EP lead (RAO view). (*B*) Coronary sinus venography (LAO view) showing no residual posterolateral CS branch. (*C*) Final LBBAP lead position (RAO view). (*D*) Pre-TLE paced ECG. (*E*) Final LBBAP ECG. CRT-D, cardiac resynchronization therapy defibrillator; CS, coronary sinus; EP, epicardial pacing; LAO, left anterior oblique; LBBAP, left bundle branch area pacing; RAO: right anterior oblique; TLE, transvenous lead extraction.

Our study underscores the reliability of LBBAP as a valuable alternative to EP in patients requiring CRT-D extraction and reimplantation (*Figure 1*). Based on our experience, LBBAP demonstrated several advantages, including (i) reduced procedure duration, (ii) lower radiologic exposure, (iii) shorter QRS duration, and (iv) lower pacing thresholds.

The study groups consisted of a real-world, homogeneous population of patients with previous heart failure with reduced ejection fraction. The mean left ventricular ejection fraction of 40.6% at the time of admission reflected the beneficial effects of ongoing anti-remodelling therapy, reinforcing the need for CRT preservation. Transvenous lead extraction has become safer in recent years due to technological innovations in less traumatic tools and increased operator expertise.^9^ Our results align with this trend, reporting only 2 complications out of 17 cases of high surgical risk (12%) identified by EROS score. Overall, skin-to-skin time and radiologic exposure were significantly lower in the LBBAP group. This finding can be attributed to technical reasons related to the shorter implantation time required by LBBAP and the increased difficulty in selecting viable remaining CS branches after extraction. As in our experience, a recent study suggested the feasibility and safety of right-sided LBBAP.^10^ The higher pacing thresholds of replaced epicardial leads compared to LBBAP highlight the need for operators to find a balance between selecting a viable CS branch and ensuring adequate electrical parameters. The LBBAP group demonstrated a significant improvement in ventricular conduction compared to EP, as evidenced by overall QRS time and delta QRS concerning the pre-operative ECG. Our study provides promising results on post-TLE LBBAP safety and feasibility that warrant confirmation in further trials.

## Data Availability

The data that support the findings of this study are available on request from the corresponding author.
